# Comparative Molecular Microbial Ecology of the Spring Haptophyte Bloom in a Greenland Arctic Oligosaline Lake

**DOI:** 10.3389/fmicb.2012.00415

**Published:** 2012-12-17

**Authors:** Susanna Theroux, Yongsong Huang, Linda Amaral-Zettler

**Affiliations:** ^1^Marine Biological Laboratory, Josephine Bay Paul Center for Comparative Molecular Biology and EvolutionWoods Hole, MA, USA; ^2^Department of Geological Sciences, Brown UniversityProvidence, RI, USA

**Keywords:** haptophytes, pyrosequencing, alkenones, lake bloom, arctic

## Abstract

The Arctic is highly sensitive to increasing global temperatures and is projected to experience dramatic ecological shifts in the next few decades. Oligosaline lakes are common in arctic regions where evaporation surpasses precipitation, however these extreme microbial communities are poorly characterized. Many oligosaline lakes, in contrast to freshwater ones, experience annual blooms of haptophyte algae that generate valuable alkenone biomarker records that can be used for paleoclimate reconstruction. These haptophyte algae are globally important, and globally distributed, aquatic phototrophs yet their presence in microbial molecular surveys is scarce. To target haptophytes in a molecular survey, we compared microbial community structure during two haptophyte bloom events in an arctic oligosaline lake, Lake BrayaSø in southwestern Greenland, using high-throughput pyrotag sequencing. Our comparison of two annual bloom events yielded surprisingly low taxon overlap, only 13% for bacterial and 26% for eukaryotic communities, which indicates significant annual variation in the underlying microbial populations. Both the bacterial and eukaryotic communities strongly resembled high-altitude and high latitude freshwater environments. In spite of high alkenone concentrations in the water column, and corresponding high haptophyte rRNA gene copy numbers, haptophyte pyrotag sequences were not the most abundant eukaryotic tag, suggesting that sequencing biases obscured relative abundance data. With over 170 haptophyte tag sequences, we observed only one haptophyte algal Operational Taxonomic Unit, a prerequisite for accurate paleoclimate reconstruction from the lake sediments. Our study is the first to examine microbial diversity in a Greenland lake using next generation sequencing and the first to target an extreme haptophyte bloom event. Our results provide a context for future explorations of aquatic ecology in the warming arctic.

## Introduction

Oligosaline lakes (salinity 0.5–5 ppt) develop in polar regions near ice sheets where evaporation exceeds precipitation and provide a unique habitat apart from the more common glacially derived freshwater lakes. These high latitude lakes serve as sensitive indicators of the ecosystem response to global climate change (Quayle et al., 2002; Marchetto et al., 2004) as their low salinity reflects small changes in hydrological balance. In the past decade alone, southwestern Greenland has undergone marked warming, and major warming is predicted for the future (Bennike et al., 2010). Microbial surveys targeting the 18S ribosomal RNA (rRNA) gene have revealed previously unknown diversity in microbial eukaryotes lineages such as cryptomonads, katablepharids, dinoflagellates, and Perkinsea (Slapeta et al., 2005; Logares et al., 2007; Shalchian-Tabrizi et al., 2011). However, haptophyte algae have been largely absent from these studies, potentially the result of naturally low haptophyte abundances in the environments selected such as deep sea habitats or anoxic lakes (Stoeck et al., 2009, 2010; Edgcomb et al., 2011; Pawlowski et al., 2011; Shalchian-Tabrizi et al., 2011). The GC-rich haptophyte genomes may also hinder amplification reactions that use universal primer sets (Moon-van der Staay et al., 2001; Liu et al., 2009; Stoeck et al., 2010). In this study, we targeted the haptophyte-rich waters of an arctic oligosaline lake spring bloom event to shed light on the microbial diversity of these unique ecosystems.

Lake BrayaSø in southwestern Greenland experiences a seasonal haptophyte bloom approximately 2 weeks after ice-off (D’Andrea et al., 2011). These haptophyte blooms result in exceptional abundances of alkenones in BrayaSø sediments (82 mg/g total organic carbon, D’Andrea and Huang, 2005) that provide the first quantitative temperature record for the past 5000 years for southwestern Greenland (D’Andrea et al., 2011). Only a few species of haptophyte algae, in the order Isochrysidales, produce alkenone lipids. These species and their alkenone lipids have been extensively studied in marine environments, where alkenones are preserved in marine sediments as a record of sea surface temperature back through time (Volkman et al., 1980; Marlowe et al., 1984; Brassell et al., 1986; Prahl and Wakeham, 1987; Müller et al., 1998; Conte et al., 2006). The endeavor to extend this alkenone-based proxy to the continents has resulted in pan-continental surveys of lake sediments and waters for alkenone-producing haptophyte algae. The use of haptophyte-specific primers targeting environmental DNA has revealed considerable diversity in lake-dwelling haptophytes (Coolen et al., 2004; D’Andrea et al., 2006; Theroux et al., 2010). However, these haptophytes are largely absent in molecular surveys targeting universal genes and microbial diversity studies of haptophyte blooms are non-existent.

Nutrient loading and seasonal irradiance levels are known to trigger marine and estuarine haptophyte blooms (Tyrrell and Merico, 2004) that occur across latitudes in both cold and coastal regions (Brown and Yoder, 1994). Increasing global temperatures will result in longer ice-free periods in arctic lakes and an increase in runoff from thawing tundra catchments, undoubtedly affecting the annual haptophyte bloom events. Given the significance of haptophyte algae in aquatic ecosystems, their absence in previous datasets, and the desire to anticipate their future response to global climate change, the objectives of our study were twofold: (1) to sequence a haptophyte-rich environment with a universal molecular approach; and (2) to produce a benchmark species survey for an arctic oligosaline lake during the spring bloom. Using high-throughput pyrotag sequencing, we targeted both bacterial and eukaryotic communities from two separate years to evaluate the consistency of the bloom-associated microbial populations. Our study provides an important baseline to contrast future BrayaSø microbial community change during its ice-free period in anticipation of a warmer Arctic possessing longer ice-free periods.

## Materials and Methods

### Site description

**Figure 1:**
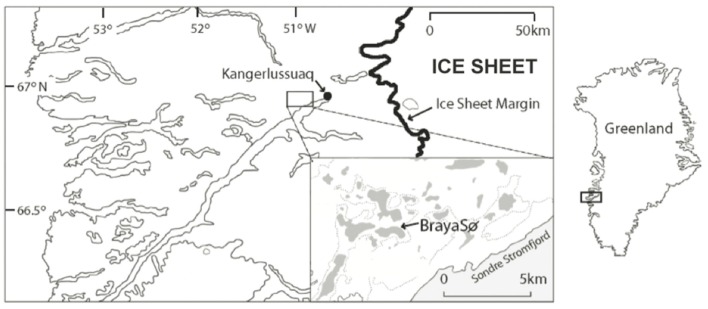
**Site map showing Lake BrayaSø in the Kangerlussuaq region, Greenland**. Modified from D’Andrea et al. (2006).

The Kangerlussuaq region of Southwestern Greenland lies at the head of the Søndre Strømfjord, 150 km from the ocean outlet, and has a series of saline lakes that provide ideal locations of paleoclimate reconstruction due to their anoxic hypolimnions and excellent preservation of lake sediments (Figure 1). Lake BrayaSø (66.99°N, −51.01°W) is a meromictic, oligosaline lake (salinity 2–3) that has an ice cap from September to late May. The surface area of Lake BrayaSø is approximately 72 hectares, with a maximum depth of 24 m. The dominant salts are NaCO_3_, NaHCO_3_, and MgHCO_3_, which are delivered to the lakes via aeolian transport from nearby sand sheets and input from erosion within the lake catchments (Anderson and Brodersen, 2001). The bedrock in the region is granodioritic gneiss with occasional ultrabasic intrusions (Heggen et al., 2010). The climate is low-Arctic continental with >500 mm/year of precipitation and a mean annual temperature at Kangerlussuaq of −6°C (Heggen et al., 2010). The lake is calcium-depleted relative to fresh lakes of the region due to CaCO_3_ precipitation, and the dominant cations are Na^+^ > Mg^2+^ > K^+^ > Ca^2+^ (D’Andrea, 2008). Total nitrogen is approximately 803 μg/L and total phosphorus is approximately 9 μg/L (Brutemark et al., 2006). Dissolved organic carbon is approximately 90 mg/L (Anderson et al., 2009). Alkenone lipids are present in the sediments of Lake BrayaSø (D’Andrea and Huang, 2005) and sediment trap data indicated the annual haptophyte bloom in the lake occurs approximately 2 weeks after ice-off (D’Andrea, 2008).

### Water column

**Figure 2:**
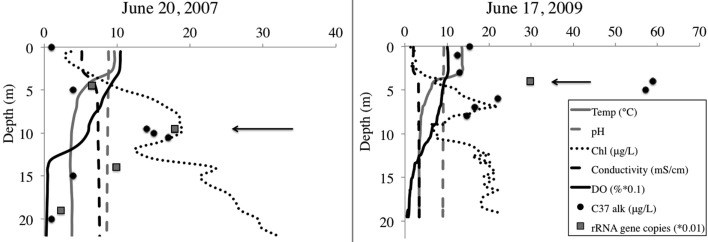
**BrayaSø water column**. Left panel shows 2007 sampling profile, and right panel shows 2009 sampling profile. Arrows denote sample depth for the samples we sequenced Dissolved oxygen (DO) is decreased one order of magnitude and haptophyte rRNA gene copy number is decreased two orders of magnitude to plot along the same axis.

**Figure 3:**
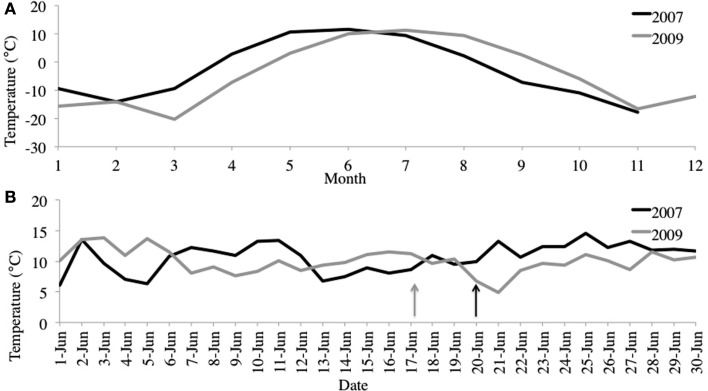
**(A)** June average air temperatures for 2007 and 2009. **(B)** Average monthly air temperatures for 2007 and 2009. Station data from Kangerlussuaq, Greenland (67.017°N, −50.700°W). http://www.ncdc.noaa.gov/oa/ncdc.html. Arrows denote sampling dates.

We selected samples for pyrosequencing based upon chlorophyll and alkenone concentrations in the water column, choosing the 10-m depth for 2007 and 4-m depth from 2009 (Figure 2). For 2007, 10-m corresponded to the peak in chlorophyll and alkenone concentrations. For 2009, 4-m depth was slightly above the chlorophyll maximum and coincided with the alkenone concentration peak (Figure 1). The June average temperature for each sampling day in 2007 and 2009 was 11.2 and 9.9°C, respectively (Figure 3A). The average daily air temperature for June 2007 was 10.7°C and for June 2009 was 10°C (Figure 3B).

### Water sampling

The bloom event occurred in BrayaSø, Greenland in June of 2007 and 2009. For both years, we analyzed a sample collected during the first week of the haptophyte bloom. We collected geochemical data using a YSI Sonde (OH, USA) equipped with probes to measure temperature, conductivity, dissolved oxygen, and chlorophyll a fluorescence. At 1-m intervals, we collected water with a Van Dorn water sampler and preserved these samples for alkenone and genomic DNA analysis. For alkenone analysis, we filtered 1 L of water onto a pre-combusted (550°C) GF/F 0.7 μm, 47 mm glass filter, and kept it frozen until analysis. For DNA analysis, we filtered a separate liter of lake water onto a 0.2 μm Sterivex™ filter (Millipore, Billerica, MA, USA), flooded the filter with Puregene lysis buffer (Qiagen, Valencia, CA, USA), and froze it at −20°C until processing. We selected samples for sequencing based on maximum alkenone concentrations.

### Lipid analysis

Alkenone extraction was after D’Andrea and Huang (2005). Alkenone samples and DNA samples were sourced from the same water sample. We freeze-dried and homogenized samples manually. We extracted samples with nine, 1 Dichloromethane (DCM), Methanol (MeOH) using an Accelerated Solvent Extractor ASE200 (Dionex, Sunnyvale, CA, USA). Extracts were separated into acid and neutral fractions using a solution of DCM, Isopropyl alcohol 2:1 (v/v). The neutral fraction was further separated into aliphatic (hexane elution), ketone (DCM), and alcohol (ethyl acetate:hexane 1:3) fractions using a flash silica gel column. The DCM fraction was analyzed using an Agilent 6890plus Gas Chromatograph Flame Ionization Detector (GC-FID) (Santa Clara, CA, USA) for quantification. Chromatograms were compared to previously reported alkenone standards and their GC retention times (de Leeuw et al., 1980; Marlowe et al., 1984). Alkenone concentrations were determined from GC-FID analysis of the ketone fractions based on an internal C_36_ alkane standard.

### DNA extraction

We extracted Sterivex™ filters using a Qiagen Puregene Cell Kit (Venlo, Netherlands) according to the manufacturer’s instructions. Genomic DNA was polyethylene glycol (PEG) purified (LaMontagne et al., 2002) to remove proteins and other contaminants that inhibit PCR reactions. DNA was suspended in PEG at 4°C overnight, centrifuged, and the pellet rinsed with ethanol. The DNA was resuspended in DNA hydration solution (Qiagen). We quantified total extracted genomic DNA yields using a NanoDrop nucleic acid spectrophotometer (Thermo Scientific, Wilmington, DE, USA) to ensure they were RNA-free.

### Quantitative polymerase chain reaction

Purified DNA extracts were also subjected to real-time quantitative polymerase chain reaction (qPCR) to gauge haptophyte cell concentrations with depth and ensure that the sample selected for sequencing was at the point of highest haptophyte cell concentration in the water column. We performed the qPCR reaction using 18S rRNA gene haptophyte-specific primers Prym-429F (5′-GCG CGT AAA TTG CCC GAA-3′; *T*_m_ = 65°C), and Prym-887R (5′-GGA ATA CGA GTG CCC CTG AC-3′; *T*_m_ = 62°C) (Simon et al., 2000; Coolen et al., 2004). These primers yield an amplicon that is approximately 463 bp in size. These primers have previously been screened for specificity: the forward primer Prym-429F is 100% specific for Haptophyta order Prymnesiales and matched 93% of orders Coccosphaerales, Isochrysidales, Prymnesiales, the genus *Pleurochrysis*, as well as unclassified haptophytes (Coolen et al., 2004). The reverse primer is specific to Prymnesiophyceae (Simon et al., 2000). We further confirmed primer specificity using the ARB probematch tool in SILVA ARB database v111 (Pruesse et al., 2007). This SSU reference database contains 739,633 high quality 16S/18S rRNA gene sequences. The Prym429F primer matched 72% of full-length 18S rRNA gene haptophyte sequences with two mismatches, and no non-haptophyte sequences. The Prym88R primer returned 99.3% of haptophyte sequences with one mismatch and no non-haptophyte sequences.

The qPCR reactions were run in triplicate, including a no-template control, on an Applied Biosystems StepOnePlus™ Real-Time PCR System (Foster City, CA, USA), using a SYBR Green I assay. We also ran a positive control of *Isochrysis galbana* DNA extracted from a culture with cell concentrations at 1.5 × 10^6^ cells/ml. The *C*_q_ for each sample had a deviation of less than 0.5. Each 20 μl reaction contained 7.2 μl of sterile water, 10 μl of KAPA SYBR® FAST Universal 2× qPCR Master Mix (Woburn, MA, USA), 0.4 μl each of the forward and reverse primers (0.2 μM) and 2 μl of template DNA. Template DNA ranged in concentration from 2 to 10 ng/μl. The qPCR cycling program was after Coolen et al. (2009) and consisted of 38 cycles of denaturation at 94°C for 30 s, annealing at 62°C for 40 s, primer extension at 72°C for 60 s, a photo step of 80°C for 20 s. We used between 10^1^ and 10^6^ copies (10-fold dilution series) of linearized plasmids containing the 18S rRNA gene of *Isochrysis galbana* CCMP1323 as the external standard to calibrate the copy numbers of haptophyte RNA genes in the BrayaSø water samples. Our standard curve was established using four points of the diluted standard, with an R^2^ value of >0.999 and slope of −3.991. Our reaction efficiency was 78.1%. We used StepOne Software version 2.2 (Applied Biosystems) to analyze our results. These conditions are reported in accordance with the Minimum Information for Publication of Quantitative Real-Time PCR Experiments (MIQE) guidelines (Bustin et al., 2009).

### Pyrosequencing

We performed genomic DNA amplifications using eukaryotic and bacterial-specific primers targeting the V9 (Amaral-Zettler et al., 2009) or V6–V4 (Morrison and Sogin, in preparation) regions, respectively. Eukaryotic sequences were generated using a Genome Sequencer FLX (Roche, Switzerland) with the GS-LR70 long-read sequencer kit at the Marine Biological Laboratory Keck Sequencing Facility. Amplifications and sequencing for eukaryotic sequences were after Amaral-Zettler et al. (2009). We sequenced the V6–V4 hypervariable region of the bacterial 16S rRNA gene using bacterial primers 515F and 1046R on a Roche GS FLX pyrosequencer using GS FLX Titanium Series reagents (Roche Diagnostics, Basel, Switzerland) following manufacturer’s protocols. Sequences were trimmed and screened for quality after Huse et al. (2007). To assign taxonomy to the remaining quality-controlled tags, we used the Global Alignment for Sequence Taxonomy (GAST) algorithm (Huse et al., 2008). Tag sequences were grouped into Operational Taxonomic Units using SLP-PWAL (refer to Huse et al., 2010), with bacterial sequences clustered at 3% and eukaryotic sequences clustered at 6%. Venn diagrams were constructed using BioVenn (Hulsen et al., 2008). Bacterial diversity estimates were calculated using EstimateS v8.2.0 (Colwell, 2005) and CatchAll (Bunge, 2011). The open source Investigation/Study/Assay (ISA) (Sansone et a., 2012) metadata-tracking framework was used to curate the datasets and format them for submission to the NCBI SRA database. All sequences have been deposited in the NCBI Sequence Read Archive (SRA) under the SRA number SRA059384, and are MIMARKS compliant (Yilmaz et al., 2011).

## Results and Discussion

### Water column

Both 2007 and 2009 samples were selected from the first week of the 2-week haptophyte bloom. In both years, secchi depth was 5 m indicating the photic zone terminated at approximately 10–12.5 m depth. The alkenone peak in 2007 corresponded to the oxycline at 10 m depth, whereas the alkenone peak in 2009 was at the thermocline.

Quantitative PCR analysis confirmed that the water sample from peak alkenone depth corresponded to peak haptophyte cell numbers (Table 1, Figure 2). The structure of the water column between the 2 years was markedly different, with chlorophyll peaking at 10 m depth in 2007 and about 6.5 m depth in 2009. Alkenone concentrations peaked at 15 μg/L in 2007 and about 59 μg/L in 2009. Correspondingly, rRNA gene copies peaked at 2146 copies/mL in 2007 and 9898 copies/mL in 2009 (Figure 1). This equates to approximate cellular alkenone concentrations of 6–7 ng/cell if the 18S rRNA gene copies occur singularly or 3–3.5 ng/cell if there are two copies of the 18S rRNA gene in these haptophytes. This is on par with previously observed cellular alkenone concentrations in lacustrine haptophytes of 0.009–2 pg/cell (Marlowe et al., 1984; Versteegh et al., 2001). Given that Lake BrayaSø has the highest sedimentary concentrations of alkenones ever reported (D’Andrea and Huang, 2005), the high cellular concentrations as estimated by our qPCR analysis is not surprising. Our results also agree with observations by Boere et al. (2011) that alkenone concentrations can serve as a proxy for haptophyte cell numbers.

**Table 1:** **BrayaSø water column C_37_ alkenone concentration and haptophyte rRNA gene copy number**.

Date	Water depth (m)	[C_37_] (μg/L)	Haptophyte rRNA gene copies/mL
6/20/07	5	4	649
6/20/07	10*	15	1,788
6/20/07	14	4	986
6/20/07	20	1	230
6/17/09	4*	59	2,969
*Isochrysis galbana control*			1,493

Asterisks (*) denote samples chosen for pyrosequencing.

### Bacterial community diversity

**Figure 4:**
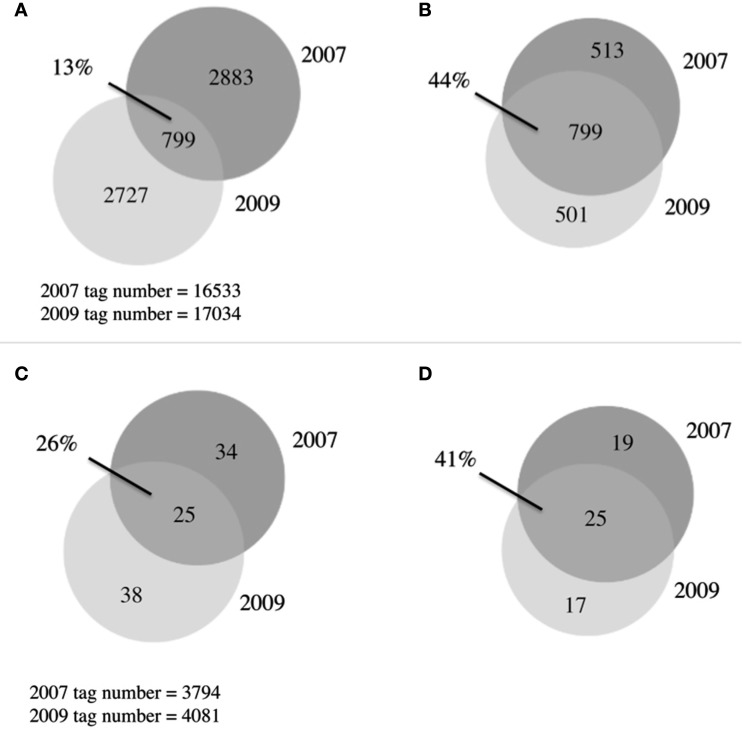
**Venn diagrams of microbial communities**. **(A)** Overlap between 2007 and 2009 bacterial communities, OTUs defined at 97% similarity **(B)** As in **(A)** with singletons removed **(C)** overlap between 2007 and 2009 eukaryotic communities, OTUs defined at 94% similarity, **(D)** as in **(C)** with singletons removed.

A total of 6,409 bacterial OTUs were observed between 2007 and 2009 (Figure 4A). The 2 years had comparable OTU yields, 2883 from 2007 and 2727 from 2009, yet shared only 13% of their OTUs. This overlap in OTUs was surprisingly low, considering reports from other arctic lake surveys demonstrating up to 73% overlap in bacterial community membership (Crump et al., 2003). When singletons were ignored, this overlap increased to 44% (Figure 4B), indicating a third of the taxon differences came from the rarest members of the community. Our study revealed much greater bacterial diversity at the phylum level, 25 phyla, than previous studies from freshwater and oligosaline lakes on the Tibetan plateau that identified only 13 phyla (Liu et al., 2010). The estimates of alpha diversity of the bacterial community generated species richness estimates with overlapping confidence bounds (Table 2), demonstrating that our similar OTU yields reflected the similar alpha diversity or richness during the 2 years.

**Table 2:** **Sequencing summary, OTU distributions, and diversity estimates**.

	Bacteria		Eukaryota	
	2007	2009	2007	2009
Sequenced tags	16 533	17 034	1 554	2 795
Total observed OTUs	3 682	3 526	59	63
Singletons	2 679	2 604	18	25
Shared OTUs	799		25	
Shared OTUs > 1	72%		100%	
Jaccard	0.125		0.258	
Sorensen	0.222		0.41	
Morisita–Horn	0.848			
Bray–Curtis	0.527			
**RICHNESS ESTIMATES**				
CatchAll estimate	27 353 (−8148, +12 425)	18 727 (−1864, +2124)		
Chao	11 142 (−838, +945)	11 060 (−866, +979)		
ACE	20 823 (−2398, +2689)	22 639 (−2749, +3211)		

*Upper and lower confidence bounds are 95%. CatchAll: Estimate, estimated total species richness; Chao1, Chao1 estimated total species richness; ACE, “abundance-based coverage estimator” estimated total species richness*.

The Morisita–Horn index of similarity, an abundance-based distance measure of beta diversity, was 0.848 (complete overlap = 1; Table 2) indicating the most abundant species were present in both 2007 and 2009. Of the most abundant bacterial OTUs (Table 3), the notable difference between communities in 2007 and 2009 was the presence of the sulfur-oxidizing bacteria in 2009 (*Thiomicrospira*, *Sulfurovum*, *Sulfuricurvum*) and fewer Flavobacteria in 2009. This flavobacterial OTU matched environmental sequences from freshwater environments, including 100% sequence identity to a bacterium isolated during a spring phytoplankton bloom in Lake Zurich (Eckert et al., 2011) and bacteria from lakes on the Tibetan plateau (Zhang and Liu, unpublished, GenBank HM128691).

**Table 3:** **Most abundant bacterial and eukaryotic OTUs**.

	2007	2009	Total	GAST
**A. BACTERIA TAXONOMY**				
Bacteria; Actinobacteria; Actinobacteria; Actinomycetales; Sporichthyaceae	1660	1136	2796	0.0112
Bacteria; Actinobacteria; Actinobacteria; Actinomycetales; Microbacteriaceae; *Limnoluna*	1115	796	1911	0.0079
Bacteria; Proteobacteria; Betaproteobacteria; Burkholderiales; Burkholderiaceae; *Polynucleobacter*	435	660	1095	0.0054
Bacteria; Proteobacteria; Betaproteobacteria; Burkholderiales; Comamonadaceae	505	488	993	0.0066
Bacteria; Proteobacteria; Alphaproteobacteria; Rickettsiales; SAR11; *Pelagibacter*	550	292	842	0.0198
Bacteria; Bacteroidetes; Flavobacteria; Flavobacteriales; Cryomorphaceae; *Owenweeksia*	429	322	751	0.0051
Bacteria; Proteobacteria; Alphaproteobacteria; Rickettsiales; SAR11; *Pelagibacter*	390	308	698	0.0242
Bacteria; Proteobacteria; Betaproteobacteria; Burkholderiales; Alcaligenaceae	174	470	644	0.009
Bacteria; Actinobacteria; Actinobacteria; Actinomycetales; Sporichthyaceae	360	268	628	0.0145
Bacteria; Chloroflexi	177	434	611	0.0121
Bacteria; Bacteroidetes; Sphingobacteria; Sphingobacteriales; Cyclobacteriaceae; *Algoriphagus*	338	191	529	0.0114
Bacteria; Actinobacteria; Actinobacteria; Acidimicrobiales; Acidimicrobiaceae	148	317	465	0.0046
Bacteria; Bacteroidetes; Flavobacteria; Flavobacteriales; Flavobacteriaceae; *Flavobacterium*	420*	25	445	0.0066
Bacteria; Actinobacteria; Actinobacteria; Actinomycetales; Sporichthyaceae	215	228	443	0.0122
Bacteria; Proteobacteria; Gammaproteobacteria; Thiotrichales; Piscirickettsiaceae; *Thiomicrospira*	5	432*	437	0.0334
Bacteria; Proteobacteria; Epsilonproteobacteria; Campylobacterales; Helicobacteraceae; *Sulfurovum*	0	424*	424	0.0344
Bacteria; Actinobacteria; Actinobacteria; Acidimicrobiales; Iamiaceae; *Iamia*	254	145	399	0.0064
Bacteria; Actinobacteria; Actinobacteria; Actinomycetales; Microbacteriaceae	142	212	354	0.0086
Bacteria; Actinobacteria; Actinobacteria; Actinomycetales; Sporichthyaceae	190	110	300	0.0099
Bacteria; Actinobacteria; Actinobacteria; Acidimicrobiales; Acidimicrobiaceae	102	182	284	0.0058
Bacteria; Actinobacteria; Actinobacteria; Nitriliruptorales; Nitriliruptoraceae; *Nitriliruptor*	74	171	245	0.0174
Bacteria; Proteobacteria; Epsilonproteobacteria; Campylobacterales; Helicobacteraceae; *Sulfuricurvum*	0	237*	237	0.0184
Bacteria; Firmicutes; Erysipelotrichi; Erysipelotrichales; Erysipelotrichaceae	90	135	225	0.1072
Bacteria; Bacteroidetes; Flavobacteria; Flavobacteriales; Flavobacteriaceae; *Lutibacter*	56	162	218	0.0102
Bacteria; Bacteroidetes; Sphingobacteria; Sphingobacteriales; Cytophagaceae; *Adhaeribacter*	116	99	215	0.0389
Bacteria; Bacteroidetes; Sphingobacteria; Sphingobacteriales	127	87	214	0.0219
Bacteria; Bacteroidetes; Sphingobacteria; Sphingobacteriales; Sphingobacteriaceae; Sphingobacteriaceae; *Pedobacter*	146	68	214	0.005
Bacteria; Firmicutes; Erysipelotrichi; Erysipelotrichales; Erysipelotrichaceae	99	107	206	0.0422
Bacteria; Proteobacteria; Betaproteobacteria; Methylophilales; Methylophilaceae	52	149	201	0.0074
Bacteria; Bacteroidetes; Flavobacteria; Flavobacteriales; Flavobacteriaceae; *Flavobacterium*	49	129	178	0.0267
Bacteria; Planctomycetes; Planctomycetacia; Planctomycetales; Planctomycetaceae; *Gemmata*	9	163*	172	0.0058
Bacteria; Verrucomicrobia; Verrucomicrobiae; Verrucomicrobiales; Verrucomicrobiaceae	9	159*	168	0.0133
Bacteria; Actinobacteria; Actinobacteria; Actinomycetales; Sporichthyaceae	97	66	163	0.0421
Bacteria; Proteobacteria; Betaproteobacteria; Burkholderiales; Burkholderiaceae; *Polynucleobacter*; cosmopolitanus	144*	14	158	0.0159
Bacteria;Planctomycetes;Planctomycetacia; Planctomycetales;Planctomycetaceae;*Pirellula*	71	70	141	0.0273
**B. EUKARYOTE TAXONOMY**				
Eukaryota; stramenopiles; Bacillariophyta; Fragilariophyceae	1518	929	2447	0.0059
Eukaryota; Alveolata; Ciliophora	864	597	1461	0.0577
Eukaryota; stramenopile	640	262	902	0.1185
Eukaryota; Metazoa; Arthropoda	0	897*	897	0.0189
Eukaryota; Viridiplantae; Chlorophyta; Chlorophyceae; Chlamydomonadales	10	501*	511	0.01
Eukaryota; Alveolata; Ciliophora	35	185	220	0.0189
Eukaryota; Alveolata; Ciliophora	1	181*	182	0.0141
Eukaryota; Haptophyceae	155	16	171	0.0179
Eukaryota; Katablepharidophyta; Katablepharidaceae	120	41	161	0.1036
Eukaryota; Viridiplantae; Chlorophyta; Chlorophyceae; Chlamydomonadales	33	110	143	0.0071
Eukaryota; Alveolata; Ciliophora	70	60	130	0.1668
Eukaryota; Cryptophyta	99*	8	107	0.0107
Eukaryota; Alveolata	1	65*	66	0.0856
Eukaryota; stramenopiles; Chrysophyceae	0	47*	47	0.0539
Eukaryota; stramenopiles; Chrysophyceae	29	16	45	0.0287

Bacterial OTUs defined at 97% similarity, eukaryote OTUs defined at 96% similarity. Asterisk (*) denotes OTUs with a relative abundance difference greater than one order of magnitude. GAST, average distance between OTU and Global Alignment for Sequence Taxonomy reference sequences. **(A)** The 35 most abundant bacterial tags, ranked by total relative abundance. **(B)** The 15 most abundant eukaryote tags, ranked by total relative abundance.

While Lake BrayaSø is oligosaline, its bacterial community resembled previously reported freshwater environments in addition to high-altitude environments. The bacterial OTUs were dominated by Actinobacteria, which are known to occur ubiquitously in terrestrial and aquatic ecosystems (Embley and Stackebrandt, 1994) and can dominate lake epilimnia (Newton et al., 2011). The most abundant actinobacterial OTU matched environmental sequences from Lake Taihu (China) and other freshwater lakes with 100% identity. The second most abundant phylum represented, the betaproteobacteria, occurs more commonly in freshwater environments than marine (Nold and Zwart, 1998) and represents the most abundant bacteria in glacial meltwater communities (Cheng and Foght, 2007). Overall, the most abundant bacterial taxa (Table 3) matched sequences from other freshwater environments, and resembled that of high-altitude lakes from the Tibetan plateau in the abundance of Actinobacteria, alpha- and beta-Proteobacteria (Xing et al., 2009; Liu et al., 2010). Lakes at high-altitude experience similar environmental pressures as lakes at high latitude, including oligotrophy, low temperature, and high UV radiation in the surface waters; the similarity in their bacterial communities suggests these particular phyla can withstand harsh environmental conditions across latitudes.

Toolik Lake in Alaska experiences an increase in primary and bacterioplankton production in the first month of spring as melting snow increases organic matter and nutrient transport into the lake and allows for an increase in sunlight reaching the water column (Hobbie et al., 1983; Crump et al., 2003). A similar trend is observed on the western shelf of the Antarctic peninsula, where seasonal melting dictates irradiance levels, mixed layer depth, and organic carbon availability (Montes-Hugo et al., 2010), with an increase in primary production resulting in an increase in bacterial production. Given the increasing global temperatures, we anticipate an increased supply of organic matter into Lake BrayaSø and thus an increase in bacterioplankton production. An increasing freshwater input as a result of melting arctic tundra may affect the local hydrological balance enough to freshen Lake BrayaSø and shift the microbial community further toward one of a more freshwater composition.

### Eukaryotic community diversity

Previous work in Lake BrayaSø identified only 11 eukaryotic phyla (Brutemark et al., 2006); using high-throughput sequencing we were able to identify nine times more phyla, including picoplankton that were undetectable with the previous visual identification methods (Table 3). A total of 97 eukaryotic OTUs were observed between 2007 and 2009, with an overlap between the 2 years of only 26% (Figure 4C; Table 2). When singletons were ignored, this number increased to 41% (Figure 4D). The eukaryotic community was dominated by diatom and ciliate OTUs in spite of the presence of a haptophyte “bloom.” Diatoms are known to have high copy numbers of their 18S rRNA genes, which may be the cause of the high abundance of their OTUs (Zhu et al., 2005; Not et al., 2008), as are alveolates which range up to 9,000 copies/cell (Prescott, 1994). Haptophyte 18S rRNA gene copy numbers are estimated at 2–3 copies/cell (Zhu et al., 2005) and our qPCR analysis using an *Isochrysis galbana* standard yielded approximate 18S rRNA gene copy number are estimated at 2–3 copies per cell (Zhu et al., 2005) and our qPCR analysis using an *Isochrysis galbana* standard yielded approximate 18S rRNA gene copy number at 1 copy per cell (Table 1). Given the high ciliate and diatom tag sequences, these patterns in eukaryotic community structure likely reiterate a cautionary note on the interpretation of abundance data for 18S rRNA gene studies, although these concerns can be minimized when comparing intra-species abundances.

The eukaryotes present in BrayaSø were typical of freshwater meso- and eutrophic environments. Diatom-related OTUs were the most abundant tags we recovered in both 2007 and 2009 (Table 3). The most abundant diatom OTU represented 31% of all eukaryotic tag sequences, and shared 100% sequence identity with araphid diatoms from fresh and brackish water. The second most abundant OTU in 2007 was assigned a ciliate taxonomy that matched environmental sequences from floodplain soil and an ephemeral pond to 96 and 95%, respectively. In contrast, the second most abundant tag in 2009 matched a metazoan, and shared 100% sequence identity with the copepod *Leptodiaptomus moorei* (GenBank AY339154). This metazoan was notably absent in the 2007 dataset. Other OTUs present in >10-fold higher abundance in 2009 versus 2007 included *Chlamydomonas*, an unidentified environmental ciliate, an alveolate, and a chrysophyte (Table 3). Of the most abundant eukaryotes, an unidentified ciliate had the greatest average GAST distance of 0.1668 (Table 3) and is likely a novel species. The similarity of protistan lineages to other freshwater environments confirms the results from other studies increasingly showing the distinction between marine and freshwater communities (Logares et al., 2007).

Haptophytes comprised only 3% of the eukaryotic community in 2007 and 0.4% in 2009 (Table 3). Despite high levels of recorded alkenones at the depth of sampling, we recovered only one type of haptophyte OTU in both years albeit in much greater abundance in 2007 than 2009 (Table 3). The most abundant tag in this OTU cluster shared 100% identity with a previously sequenced 18S rRNA gene from the BrayaSø water column (GenBank HQ446272; Theroux et al., 2010). We detected identical haptophyte V9 tag reads to this BrayaSø OTU from Toolik Lake, AK, USA (Crump et al., 2012) and Plum Island, MA, USA (Amaral-Zettler, personal observation) but nowhere else in the VAMPS global database (http://vamps.mbl.edu). Singularity of the haptophyte population impacts the ability to use alkenone-derived paleoclimate records from a site; the presence of multiple haptophyte species during a bloom could jeopardize the reliability of the alkenone record. The occurrence of a single haptophyte OTU in both 2007 and 2009 samples is encouraging for the use of this environment as a paleoclimate archive; alkenone-based temperature reconstructions would therefore only require a single temperature calibration for the single haptophyte species present.

The alkenone concentrations at peak alkenone depths in the water column were 15 μg/L in 2007 and 59 μg/L in 2009. Our qPCR results (Table 1) indicated that there were approximately 1700 and 2900 haptophyte cells/mL in the 2007 and 2009 water samples, respectively. Our qPCR results also indicated that *Isochrysis galbana* has 1–2 copies of its 18 rRNA gene/cell. The occurrence of only 155 and 16 haptophyte tags in 2007 and 2009, respectively, suggested that the haptophyte DNA may have been dwarfed by greater copy number diatom and ciliate 18S rRNA sequences. Empirical obstacles also may have resulted in the low haptophyte tag yield, including primer mismatches or difficulties amplifying GC-rich haptophyte DNA.

## Conclusion

Arctic lakes will undoubtedly experience shifts in microbial populations with increasing annual temperatures, prolonged ice-free periods, and thawing tundra catchments. This study is the first to examine the microbial community of an artic oligosaline lake using high-throughput sequencing, providing a deeper resolution of the microbial community structure in these rapidly changing arctic environments. Using high-throughput sequencing, we were able to detect greater phylum-richness and new phyla previously unobserved in BrayaSø, the benefit of a molecular versus microscopy-based approach. Even though BrayaSø is an oligosaline lake, both the bacterial and eukaryotic communities resembled other high latitude and high-altitude freshwater environments. The low overlap in microbial communities between the 2007 and 2009 samplings suggested large interannual variations in microbial species. However, the 2009 sample had fewer haptophyte tags but a greater abundance of other phototrophs, suggesting the functional overlap of the eukaryotic communities may be greater than the species overlap. Future studies examining microbial populations throughout the course of a spring bloom event will help resolve these temporal shifts in species abundances and functional roles.

This study is also the first to analyze a haptophyte bloom event using next generation sequencing. We generated fewer haptophyte pyrotag sequences than expected given their alkenone biomarker concentrations in the water column. Our qPCR data confirmed that haptophyte cell numbers peaked with alkenone concentrations, and also showed that high-throughput tag sequences for haptophytes did not correspond well with qPCR counts. This result serves as a reminder that the interpretation of relative abundance data using a tag sequencing approach with eukaryotes must be done so cautiously and that complimentary, haptophyte-specific qPCR provides greater detail of cell abundances. Given the depth of DNA sequencing, and the generation of over 200 haptophyte tags, we are encouraged by the presence of a single haptophyte OTU in Lake BrayaSø, and maintain that this is a worthy location for temperature reconstruction using alkenone-based proxies. Future studies throughout the haptophyte bloom event in BrayaSø will resolve the temporal shifts in microbial communities and will help decipher the communities most susceptible to increasing arctic temperatures.

## Conflict of Interest Statement

The authors declare that the research was conducted in the absence of any commercial or financial relationships that could be construed as a potential conflict of interest.
